# Interoperable Traceability in Agrifood Supply Chains: Enhancing Transport Systems Through IoT Sensor Data, Blockchain, and DataSpace †

**DOI:** 10.3390/s25113419

**Published:** 2025-05-29

**Authors:** Giovanni Farina, Alexander Kocian, Gianluca Brunori, Stefano Chessa, Maria Bonaria Lai, Daniele Nardi, Claudio Schifanella, Susanna Bonura, Nicola Masi, Sergio Comella, Fiorenzo Ambrosino, Angelo Mariano, Lucio Colizzi, Giovanna Maria Dimitri, Marco Gori, Franco Scarselli, Silvia Bonomi, Enrico Almici, Luca Antiga, Antonio Salvatore Fiorentino, Lucio Moreschi

**Affiliations:** 1 Department of Engineering, Niccolò Cusano University, 00166 Rome, Italy; giovanni.farina@unicusano.it; 2 University of Pisa, 56124 Pisa, Italy; gianluca.brunori@unipi.it (G.B.); stefano.chessa@unipi.it (S.C.); 3 Department of Economics and Business Sciences, University of Cagliari, 09123 Cagliari, Italy; mariab.lai@unica.it; 4 Department of Computer, Control and Management Engineering, Sapienza University of Rome, 00185 Rome, Italy; nardi@diag.uniroma1.it (D.N.); bonomi@diag.uniroma1.it (S.B.); 5 Computer Science Department, University of Turin, 10126 Turin, Italy; claudio.schifanella@unito.it; 6 Engineering Ingegneria Informatica S.p.A, 00144 Roma, Italy; susanna.bonura@eng.it (S.B.); nicola.masi@eng.it (N.M.); sergio.comella@eng.it (S.C.); 7 ENEA Italian National Agency for New Technologies, Energy and Sustainable Economic Development, 00123 Rome, Italy; fiorenzo.ambrosino@enea.it (F.A.); angelo.mariano@enea.it (A.M.); 8 Dipartimento di Informatica, Università degli Studi di Bari, 70126 Bari, Italy; lucio.colizzi@uniba.it; 9 Department of Information Engineering and Mathematics (DIISM), University of Siena, 53100 Siena, Italy; giovanna.dimitri@unisi.it (G.M.D.); marco.gori@unisi.it (M.G.); franco.scarselli@unisi.it (F.S.); 10 Antares Vision Group, 25039 Travagliato, Italy; enrico.almici@antaresvision.com (E.A.); lucio.moreschi@antaresvision.com (L.M.); 11 OROBIX Srl, 24121 Bergamo, Italy; luca.antiga@orobix.com (L.A.); antonio.fiorentino@orobix.com (A.S.F.)

**Keywords:** traceability, intelligent transport systems, international data space, blockchain, internet of things, agrifood

## Abstract

Traceability plays a critical role in ensuring the quality, safety, and transparency of supply chains, where transportation stakeholders are fundamental to the efficient movement of goods. However, the diversity of actors involved poses significant challenges to achieving these goals. Each organization typically operates its own information system, tailored to manage internal data, but often lacks the ability to communicate effectively with external systems. Moreover, when data exchange between different systems is required, it becomes critical to maintain full control over the shared data and to manage access rights precisely. In this work, we propose the concept of *interoperable traceability*. We present a model that enables the seamless integration of data from sensors, IoT devices, data management platforms, and distributed ledger technologies (DLT) within a newly designed data space architecture. We also demonstrate a practical implementation of this concept by applying it to real-world scenarios in the agri-food sector, with direct implications for transportation systems and all stakeholders in a supply chain. Our demonstrator supports the secure exchange of traceability data between existing systems, providing stakeholders with a novel approach to managing and auditing data with increased transparency and efficiency.

## 1. Introduction

*Traceability* is defined in [1] as the ability to access all relevant information about a product throughout its life cycle by recording its identification. In the context of business and food supply chain management, traceability refers to the systematic monitoring and recording of product movements. This encompasses the entire process, from the origin of the product to its consumption, including all relevant stages such as production, processing, distribution, and sale. Comprehensive documentation at each stage is crucial for ensuring the integrity and reliability of the supply chain.

A distinction is posited between *mandatory* and *voluntary* traceability. The former refers to the existence of legal rules requiring the retention and disclosure of information to which system actors are obliged to comply with. The latter refers to the rules that individual companies adopt to pursue their objectives.

As-known transparency, in the context of data, ensures that stakeholders possess and share comprehension of product-related information. It is characterized by the accessibility of data in their original form free from any loss, distortion, or noise that may compromise their integrity. This openness is conducive to a number of advantages for supply chains, including enhanced market efficiency, optimized information flow among stakeholders, and optimized logistics and business processes [2].

Supply chains are diverse logistics networks characterized by specific constraints that require reliable information, communication, and transportation systems. In the agri-food context, a wide range of information about goods is stored and exchanged. Traceability promotes accountability, efficiency, and compliance with regulatory standards, helping to mitigate risks, improve quality control, and increase customer confidence [3].

Transportation systems play a pivotal role in supply chains, connecting suppliers, manufacturers, distributors, and customers to ensure the seamless flow of goods. Moreover, they guarantee the integrity of goods during transit, directly influencing how products are handled and exposed to various conditions. Environmental factors such as temperature and humidity are critical for maintaining product quality during transportation. For instance, perishable goods like fruits and vegetables are susceptible to deterioration when exposed to temperature fluctuations. Maintaining optimal temperature and humidity levels prevents issues such as spoilage, mold, and mildew formation.

Transportation bottlenecks can lead to delays, increased costs, and reduced product quality. In food supply chains, delays may result in significant spoilage and waste, adversely affecting retailers and consumers. Ensuring seamless and punctual transportation is essential to maintaining product quality and meeting customer expectations. The integration of advanced tracking technologies into transportation networks enhances real-time shipment visibility, ensuring efficient delivery while complying with environmental requirements.

The integration of Internet of Things (IoT) sensors into supply chains significantly improves traceability by providing real-time data on the location, condition, and status of products and assets. Key sensors include GPS trackers, RFID tags, and chemical sensors. Integrating these sensors with existing transportation management systems (TMS) enables companies to monitor supply chains in real-time, facilitating proactive decision making critical for maintaining product quality and addressing transit disruptions [4,5]. Additionally, digital platforms and data analytics in transportation and other stages help identify bottlenecks and inefficiencies.

Implementing a traceability system across the supply chain requires a comprehensive understanding of the broader ecosystem of stakeholders and infrastructures. Establishing agreements between stakeholders is also a prerequisite for effective collaboration [6]. In the agri-food sector, traceability is particularly important. The concept of Food Traceability 4.0, as introduced by Hassoun [7], refers to smart traceability systems spanning from farm to fork. These systems leverage Industry 4.0 technologies, including blockchain, IoT, Artificial Intelligence (AI), and big data analytics [8]. These technologies are equally advantageous for integration into intelligent transportation systems.

In supply chains, multiple companies often operate their own information systems tailored to their internal business requirements. Consequently, there is an increasing need to facilitate secure data sharing, with mechanisms to govern the nature of shared data and the entities authorized to access it [9].

However, systems capable of efficiently integrating diverse data sources and management systems are often lacking. This is due to the heterogeneity and variety of available sensors, which hinder the development of fully traceable systems [10].

This paper proposes the design of a unified federated data lake that integrates information from IoT devices, data management systems, and distributed ledger technologies (DLTs) to enable interoperable traceability. Specifically, product data are exchanged across diverse systems and platforms using standardized, interoperable methods, ensuring consistent, accurate, and transparent information flow throughout the supply chain.

The METRIQA platform [11], which provides a safe and compatible environment for data interchange, exemplifies this idea. Using blockchain, artificial intelligence, and big data analytics, the platform architecture comprises elements for data storage, decision support, and user interfaces. The useful uses of interoperable traceability in fields including automated data sensing, wine production, cattle tracking, and traceability in mobility are demonstrated by four use examples. The advantages of enhanced fraud prevention and data integration are emphasized. Although producers and transformers of goods are the main focus of the use cases mentioned, the presented solution can be applied to any supply chain participant, including transportation firms. One use case is presented in detail, including the technical specifications of the METRIQA components employed. Then, alternative solutions are assessed for each used component.

## 2. Background and State of the Art

Supply chain traceability has become a significant area of research focus due to increasing global complexity, stricter regulatory requirements, and rising consumer expectations for transparency [12]. The process involves monitoring the movement of goods, materials, and information through various stages of production, transportation, and distribution. The advent of Industry 4.0 has introduced disruptive technologies that have been integrated into traceability systems across multiple sectors [3].

The agri-food industry, in particular, has seen a growing demand for transparency and safety, driven by consumers and companies seeking to enhance the perceived value of their products. Food industry operators may, in addition to mandatory disclosures, voluntarily include supplementary information such as certifications.

Several regulatory references represent legally binding directives established to safeguard public health and manage food-related risks. The *General Principles of Food Hygiene (CXC 1-1969)* [13] by the FAO/WHO outlines necessary hygiene and food safety controls at every stage of the food chain, including measures to prevent contamination and ensure traceability. *The European Union Regulations No 178/2002* [14] and *931/2011* [15] mandate that all food and feed business operators implement traceability systems capable of identifying immediate suppliers and recipients of their products, commonly known as the “one step back—one step forward” approach. The latter regulation emphasizes meticulous traceability for food of animal origin, including documentation of batch numbers, volumes, and suppliers. *The United States Food Safety Modernization Act (FSMA)* [16] requires enhanced traceability for specific high-risk foods, as listed in the Food Traceability List (FTL), to facilitate the rapid identification and removal of potentially contaminated food from the market.

Additionally, numerous normative references guide the implementation of traceability systems, establishing the basis for compliance and certification. *ISO 22005:2007* [17] outlines general principles and basic requirements for designing and implementing feed and food traceability systems. *ISO 22000 series* [18] establishes food safety management standards integrating Hazard Analysis and Critical Control Point (HACCP) principles. The *Global Traceability Standard (GTS)* [19] provides guidelines for identifying, capturing, and sharing traceability data across end-to-end supply chains.

The transportation industry faces challenges similar to those encountered by other supply chain stakeholders, including suboptimal communication due to reliance on manual data entry, lack of integrated systems for seamless information exchange, and frequent data flow gaps. Additionally, many systems lack real-time tracking capabilities [20]. These deficiencies result in cascading inefficiencies, operational delays, and financial losses across interconnected networks, undermining accurate tracking and tracing.

To address these challenges, it is essential to establish effective mechanisms for facilitating and enhancing data sharing among stakeholders. This approach mitigates information asymmetries, fosters collaboration, and enables the collection of data from different supply chain stages. Enhancing interoperability among systems is crucial for effective information exchange, optimizing operational efficiency, and delivering improved services to consumers. Digital tools such as the Internet of Things (IoT), artificial intelligence (AI), blockchain, and distributed ledger technology (DLT) can expedite this process.

The IoT plays a pivotal role in capturing real-time data throughout the supply chain, including environmental conditions, location tracking, and product handling data. Integrating IoT with other technologies facilitates end-to-end traceability [21,22].

Data analytics enables stakeholders to comprehensively examine extensive and varied datasets generated by traceability systems. This facilitates data-driven decision making, leading to optimized operations and enhanced efficiency while promoting strategic growth through informed insights [23].

Blockchain and DLT have revolutionized supply chains and intelligent transportation systems by providing transparency, security, and the immutability of supply chain information. These technologies empower stakeholders to verify and trust data without relying on a central authority. Blockchain’s immutability ensures data integrity, preventing alteration or deletion, and, thus, mitigating fraudulent activities such as mislabeling, counterfeiting, or product substitution. Its decentralized nature, combined with cryptography, enhances security and availability against cyber-attacks, protecting stakeholders’ interests. Studies have demonstrated the effectiveness of DLT in traceability systems [24,25,26].

Efforts to standardize blockchain protocols aim to facilitate seamless interoperability between disparate systems, addressing key challenges hindering the widespread adoption of blockchain technology in supply chains and the agri-food sector [27].

The integration of IoT and blockchain technologies is pivotal in developing next-generation traceability solutions for agricultural and livestock supply chain logistics. In a conventional architecture, IoT devices function as the ground-level data collection layer, while blockchain serves as the secure, shared database layer that all stakeholders rely on [28,29]. Recent frameworks adopt a multi-tier design, comprising an IoT layer (sensors, smart tags, drones, etc.) feeding data to an edge or cloud layer for initial aggregation and processing. This layer commits sanitized key events to the DLT layer for permanent record and wider accessibility [30]. This layered approach balances the high volume and velocity of IoT data with the relatively slower, costlier operations of blockchain. For instance, IoT sensors programmed to take readings at one-minute intervals may employ cloud analytics or edge computing to aggregate or filter data, recording significant events or summary metrics (e.g., daily quality reports or location changes) onto the blockchain [28].

The integration of IoT and blockchain has been demonstrated in crop production, particularly in ensuring food product traceability. IoT-tagged identifiers, such as RFID, NFC tags, or QR codes, are attached to produce at the farm gate. As each batch progresses through the supply chain, IoT devices (e.g., temperature and humidity sensors in transport containers or scanners at checkpoints) record events such as harvest time, processing steps, storage conditions, and handovers between actors. Each event is encrypted and incorporated into the blockchain as a transaction, creating a comprehensive provenance record [29].

For example, an NFC-based tagging system embeds inexpensive NFC chips in produce crates. Scanning the chip yields a product ID linking to a blockchain record containing its origin, farm location, harvesting practices, and real-time sensor data about its storage environment. At subsequent stages (packing, shipping, and retail), additional data (quality inspections, timestamps, and custody transfers) are appended to this digital record. By the time the product reaches a consumer, it carries an accessible history verifying its safe handling and origin [30].

Such systems have been piloted for coffee beans, organic vegetables, and fruit supply chains. Scanning a code on the final product enables consumers and retailers to query the blockchain for information regarding the farm-to-store journey. Preliminary studies indicate that this approach markedly enhances transparency, trust, and efficiency. Stakeholders can audit compliance with safety standards in real-time, and any anomalies (e.g., a break in the cold chain or a delay in transit) are visible and traceable to the responsible party [30].

Researchers are addressing data management challenges by combining blockchains with off-chain storage solutions. For instance, large sensor datasets or images (e.g., detailed crop condition photos captured by drones) can be stored in a distributed file system like IPFS, with only cryptographic hashes or metadata recorded on the blockchain [31]. This hybrid approach allows bulky IoT data to be shared peer-to-peer without bloating the blockchain while guaranteeing data integrity.

In crop agriculture, IoT–DLT integration is driving traceability evolution beyond basic tracking to intelligent and automated audits of farming practices and logistics. This progression aligns with sustainability and quality certifications in a verifiable manner.

The livestock sector, encompassing meat and dairy supply chains, has similarly adopted IoT and blockchain technologies for traceability and farm management. Smart livestock farming models leverage IoT sensors to monitor animal welfare and link those records to blockchain, creating a lifelong digital trail for each animal or animal product [32].

For instance, IoT-powered cattle tracking systems use GPS collars and geo-fencing to define protective zones for free-grazing cattle. Alerts are generated if animals stray beyond boundaries or approach hazards. Such geolocation events and environmental sensor readings (e.g., local temperature, water quality) are written on a blockchain, ensuring transparency and immutability. By the time an animal is sent to slaughter, the blockchain contains an immutable log of its rearing conditions, health treatments (e.g., vaccines or antibiotics), and movement history [32].

When these data accompany meat through processing and distribution, they provides strong assurances of quality and safety to processors, retailers, and end-consumers. Comprehensive traceability data on livestock, encompassing the entire supply chain from birth to the final product, significantly enhance consumer confidence in meat and dairy products.

Pilot studies on dairy supply chains (milk and cheese) demonstrate that integrating IoT sensors (e.g., for milk temperature, storage durations) with blockchain effectively maintains product integrity and detects adulteration or spoilage in a timely manner. Each milk batch’s record becomes tamper-proof, crucial in industries like dairy where mixing inputs from many farms can obscure contamination sources. Additionally, smart contracts enforce best practices: one prototype invalidates transactions and flags alerts if unauthorized medicines or out-of-range additives are logged for an animal [32].

This kind of real-time compliance auditing is nearly impossible with paper records but becomes feasible with IoT data triggering blockchain logic. Academic prototypes and industry trials (e.g., IBM Food Trust, Walmart’s pork traceability pilot in China) validate that blockchain-backed livestock traceability significantly reduces response time in animal product recalls and bolsters consumer confidence in labels like hormone-free or grass-fed by providing verifiable claims [29].

Globally, large food corporations and consortia are experimenting with DLT for livestock and poultry supply chains (e.g., tracing beef cattle, poultry farm conditions, or egg batches on blockchain platforms). Early adopters report improved credibility, efficiency, and safety in operations when food products can be instantly traced from their point of origin through all points of contact to the consumer.

Data spaces are an evolving paradigm in data sharing, first introduced in 2005 as a significant shift from centralized databases to decentralized ecosystems of heterogeneous data [33]. They are designed to facilitate secure and trustworthy data exchange while preserving the autonomy of data sources.

The European Strategy for Data envisions a unified market for data in Europe, aiming to enhance global competitiveness and ensure data sovereignty. A cornerstone of this strategy is the creation of Common European Data Spaces, which promote cross-sectoral data sharing under a framework of trust and interoperability [34].

A notable specification for data spaces is the International Data Space (IDS), which specifies the requirements for data sovereignty, trustworthiness, and monetization [35]. The IDS supports implementing various business models centered on data exchanges characterized by consistent rules, certified data providers and recipients, and trust between partners ensured by certified components and secure interactions.

The IDS specification lists the necessary elements to establish a minimally feasible data space: (i) two or more IDS connectors that can communicate data between sources and providers, (ii) a certificate authority (CA) proving X.509 certificates to enable authenticity and integrity of data exchange, and (iii) a dynamic attributes provisioning service (DAPS) [35,36] aimed at regulating information security and access control. It issues short-lived tokens (dynamic attribute tokens, DATs) that carry verified attributes about a participant or Connector. In practice, each Connector requests a digitally signed JSON web token (JWT) from DAPS to authenticate and obtain current identity attributes. This means that rather than embedding all attributes in long-lived certificates, IDS can update or revoke attributes dynamically via the DAPS without reissuing certificates [37].

Interoperability between dataspace connectors requires a protocol-agnostic foundation. To address this need, IDSA is developing a dedicated dataspace protocol—a set of specifications built on Web technologies and designed to enable interoperable data sharing with usage control. Table 1 presents the current connectors that comply with IDS standards and have reached a technology readiness level (TRL) of 5 or higher [38].

## 3. Interoperable Traceability and Use Cases

The accuracy and fluidity of information flows between supply chain participants are among the main goals of traceability procedures. Since different players may follow their own codifying protocols and utilize their own technologies, the primary obstacles to accomplishing these goals are associated with the variety of data encoding and transmission methods. The idea of *interoperability*, which is the ability of systems to share and utilize information, is used to overcome this problem. This idea has several facets, such as organizational, legal, semantic, and technical elements [39].

While achieving interoperability within a company can rely on hierarchical power, the involvement of independent actors in traceability requires the development of governance systems. It has been demonstrated that supply chains characterized by strong coordination are more likely to possess interoperable traceability systems compared to those with weak coordination. This is because the chain leader can impose its own traceability systems and technologies. For smaller firms, adopting the chain leader’s traceability system could result in a loss of autonomy, as transitioning to a different customer might require switching to another traceability system.

The idea of *interoperable traceability* was conceived to address these problems. To meet interoperability requirements, it seeks to integrate several technologies, including data spaces, DLT, IoT, and AI. In this context, we present a paradigm that facilitates the combination of data from distributed ledgers, data management software, and Internet of Things (IoT) devices within a data space. Anyone with the necessary access rights can use, integrate, improve, and transfer traceability data into their traceability systems thanks to this data space, which is made up of an instance of the International Data Space [35].

To illustrate how the developed process aligns with the identified needs, consider the following use cases that would benefit from interoperable traceability.

**Use Case 1:** ***Facilitating Information Sharing in Wine Supply Chain Monitoring Systems***

In the first use case, a wine company uses its proprietary information system to document different parts of the wine manufacturing process. The goal of this methodical technique is to guarantee the wine’s quality, originality, and traceability. Data about the vineyard, harvest, winery operations, bottling, packaging, and other phases are all included.

The producer must *create linkages with these systems* in order to share pertinent traceability data along the supply chain when selling to several wine wholesalers, each of whom has its own supply chain management (SCM) system.

For the wine maker and other stakeholders, the incompatibility of these systems presents serious difficulties. The creation of ad hoc solutions, such as custom middleware for data transformation, APIs for real-time data transmission, and modifications to handle different data formats, is frequently necessary to establish connections with these systems.

In contrast, the components of interoperable traceability, especially the data space, provide a seamless way to link these systems, enhancing efficiency and significantly reducing costs.

**Use Case 2:** ***Data Sensing to Enhance Supply Chain Traceability***

*Automated data collection* greatly improves the reliability and transparency of supply chain processes, enabling effective tracking and verification of product quality, origin, and handling.

This enhances consumer confidence and increases operational efficiency. Moreover, artificial intelligence transforms these data into valuable insights about product quality for consumers.

Imagine a field with several IoT nodes that optimize water and fertilizer consumption by continuously monitoring important soil and crop substrate factors. Important parameters, including pH, temperature, nitrogen, phosphorus, potassium, and soil moisture, are all measured by these sensors. The accuracy of the data gathered is essential because the data directly influence supply chain decision making. A back-end system receives real-time measurements, stores them safely in a database, and certifies them via a blockchain system. This method promotes reliability, guarantees data integrity, and increases transparency. Since these data serve as the basis for well-informed decisions throughout the supply chain, their security and accuracy are crucial.

Interoperable traceability technologies simplify automated data acquisition, guarantee data integrity, and support AI-assisted evaluation. By using high-quality data, farmers can grow healthier crops while reducing their impact on the environment. This not only increases the value of their products but also builds trust among consumers and retailers.

**Use Case 3:** ***Fraud Prevention and Interoperable Cattle Traceability for Food Supply Chain Elements***

The objective of how to successfully integrate interoperable traceability with fraud prevention mechanisms is brought up by Use Cases 1 and 2.

Consider a normal cow grazing in an alpine pasture while wearing a smart collar that gathers GPS data and stores it in the farmer’s information system to demonstrate this concept. As the animal enters the supply chain, this configuration offers traceability information about its grazing location. As a matter of fact, a livestock farmer can engage in fraudulent practices. For example, he/she could claim ownership of other people’s cattle and sell them as their own, or falsely claim compliance with feeding standards.

To mitigate these issues, it is important to guarantee that the GPS data produced by the collar and stored in the farmer’s system remain unaltered. This can be accomplished by using a tamper-proof collar and an integrity code for the GPS data. In this context, interoperable traceability technologies provide critical support by (I) enabling the secure sharing of data to substantiate claims, (II) ensuring the integrity and immutability of the data, and (III) providing access to AI models that assist in verifying claims.

**Use Case 3.5:** ***Extending Sensing in Interoperable Cattle Traceability to Welfare Monitoring***

Use Case 3 focuses solely on GPS sensing, while Use Case 2 leverages AI for decision support systems. Another relevant use case pertains to the growing need for uninterrupted monitoring of animal health and well-being within agricultural contexts. The implementation of such monitoring systems has been proven to facilitate the early detection of behavioral and motor abnormalities, enabling timely interventions.

Cattle traceability can be significantly enhanced by incorporating a diverse range of sensor data, including cameras and RFID tags, which can be analyzed with AI support to assess cattle welfare. Two key parameters for evaluating cattle welfare are the Locomotion Score and the Body Condition Score [40].

The *Locomotion Score* is a scale used to assess the quality of cattle gait on a scale from 1 (normal) to 5 (severe lameness). The automatic evaluation process entails the analysis of eight locomotion characteristics extracted from lateral video footage. The temporal sequence of these features is processed using a long short-term memory (LSTM) network, which estimates the score for each time window. The final score is determined by calculating the average of the scores obtained across all time windows.

The *Body Condition Score* quantifies the fat reserves of cows on a scale from 1 (emaciated) to 5 (obese). Assessment is based on zenithal imaging of the animal after identification via RFID tags. A detection model is employed to isolate the cow’s body within the frame, which is then processed by two models in parallel: a BCS classifier and a markerless identification network. Score estimation is handled by a ResNet-based convolutional neural network.

Interoperable traceability technologies would support the maintenance of a detailed history for each animal and the entire herd, the deployment of AI models, and the accountability of declared data. All of this would enable proper documentation of animal welfare over time, fostering transparency and trust across the supply chain.

**Use Case 4:** ***Sensing and Traceability in Mobility***

In the context of a production chain, transportation assumes a pivotal role in the movement of products along the logistics chain. From a traceability perspective, transportation can significantly impact the quality of the final product, particularly within agrifood supply chains. It is crucial to manage products, including processed goods, meticulously to avoid their rapid deterioration. For instance, the storage and transportation of frozen goods require strict temperature control measures to ensure their integrity and quality.

Referring to Use Cases 3 and 3.5, which focus on high-quality cattle farming with attention to health and wellness, transportation (e.g., from the farm to the slaughterhouse) can also be monitored using sensors. These sensors may include smart collars, as documented in Use Case 3, or environmental sensors, as in Use Case 3.5, now deployed in livestock trucks. In such scenarios, two key requirements remain relevant: the interoperability of sensor data collected during transport and the prevention of fraud. Sensor data collected during transportation constitute an integral component of the traceability data required to ensure the final quality of products for consumers. Consequently, the integrity of these data may be compromised by the possibility of fraudulent activity aimed at manipulating the data.

To address these challenges, the interoperable traceability solutions previously outlined should be applied. This involves adopting a data space to enable interoperability for sensor data, along with a secondary flow of integrity data automatically generated from the sensor data. The integrity data are stored in a blockchain whenever the livestock is handed over to the next actor in the supply chain, ensuring transparency and trustworthiness.

### Technological Components for Interoperable Traceability

The presented use cases provide examples of specific parts of supply chains with related needs. In this section, we briefly outline a series of technologies that have the capacity to support efficient and interoperable traceability.

We clarify the primary benefits of each technology envisioned for interoperable traceability:

- **IoT Devices and Sensors**: automate data collection, eliminating manual entry and reducing errors; measure environmental and product characteristics; enable real-time data collection; facilitate direct information exchange with IT systems.
- **Data Analytics and AI**: enhance pattern recognition; perform predictive analytics; support anomaly detection.
- **Distributed Ledger Technology (DLT)**: ensure permanent high availability of data; provide accountability; maintain data integrity.
- **Data Space**: enable interoperability; facilitate data integration; support controlled data exchanges; ensure data sovereignty.

Heterogeneous system integration relies on these four complementary pillars. IoT devices and sensors ubiquitously capture diverse data streams—from environmental measurements to machine telemetry—across multiple domains and protocols. Data analytics and AI ingest and harmonize these multimodal datasets, applying edge- and cloud-based intelligence for real-time decision support while adapting models to local contexts. Distributed ledger technology (DLT) provides a tamper-proof, decentralized trust layer, ensuring secure provenance, access control, and auditability across federated systems. Data spaces create a governed, semantic interoperability fabric that unifies cross-enterprise data exchange through common vocabularies, APIs, and consent frameworks.

Together, these technologies provide an end-to-end framework: IoT sensors feed rich, heterogeneous data; analytics and AI contextualize and refine insights; DLT underpins secure collaboration; and data spaces orchestrate governance and standardized sharing—enabling seamless integration across disparate systems.

## 4. METRIQA Digital Information Platform

As part of Italy’s contribution to the European Green Deal, which aims to achieve climate neutrality by 2050, the Italian National Research Center for Agricultural Technologies (AGRITECH) developed the METRIQA (MEasurements, TRaceabilIty, and Quality in Agri-food Chains) platform [41]. A tangible illustration of a digital information platform created to facilitate interoperable traceability in the agri-food industry is METRIQA.

Through two main channels, METRIQA plays a key role in the digital transformation of the agrifood industry in Italy. First off, by handling the complete data and information lifecycle, the platform facilitates agri-food research. This covers the intelligent environment-based management of production, storage, and analysis. During on-site trials, researchers and IoT sensors create experimental datasets. These datasets are processed by AI technologies and stored in a decentralized fashion to create cutting-edge decision support systems. Second, METRIQA provides smooth, web-like access to services and data. It also offers an open infrastructure that encourages cooperation and creativity in the agrifood industry.

**Figure 1:**
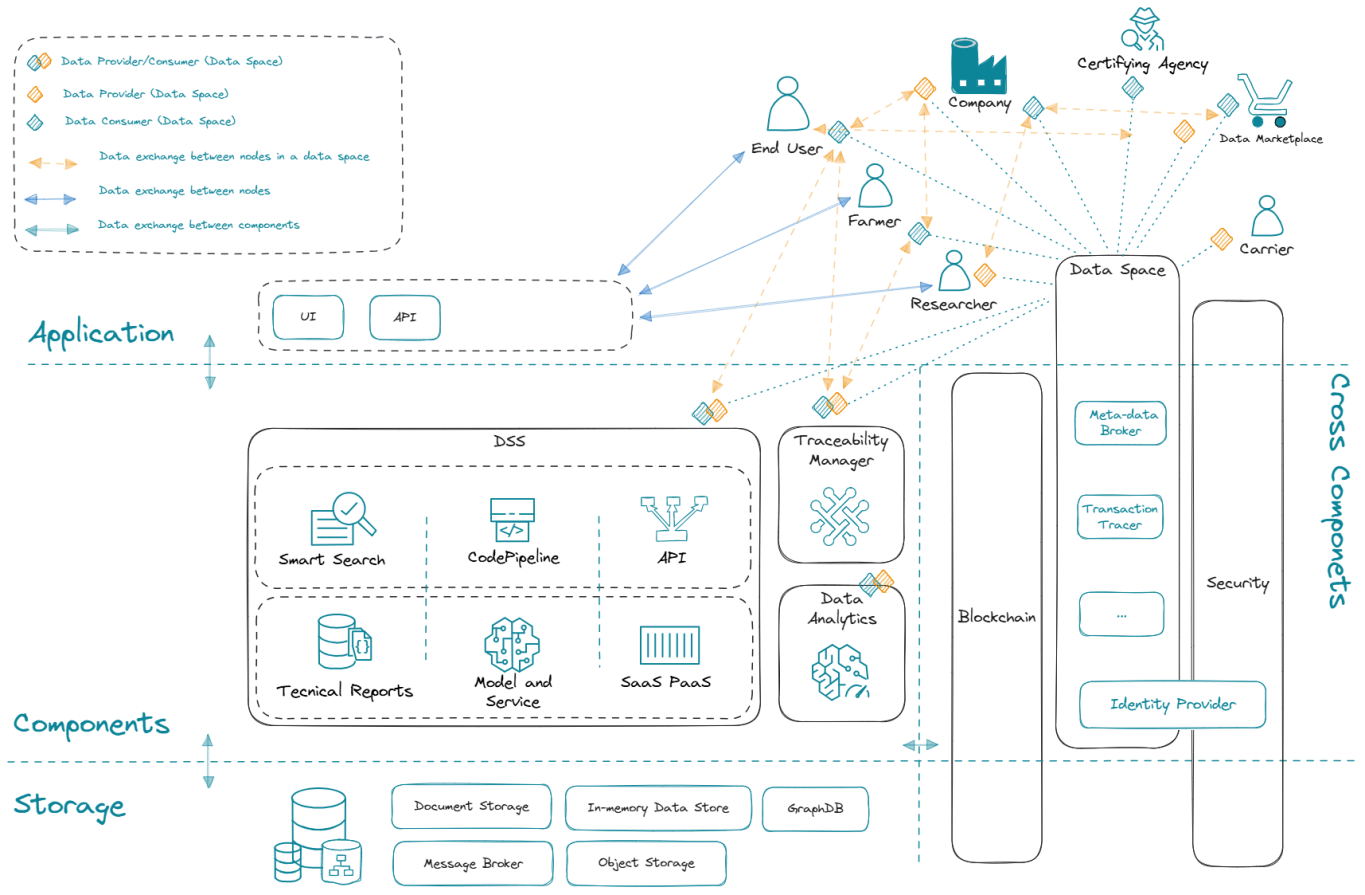
The reference architecture of the METRIQA platform.

Three levels make up the METRIQA reference architecture, which is depicted in Figure 1: Storage, Components, and Applications [34,42]. Apache Kafka is used by METRIQA as a message broker at the *Storage layer* https://kafka.apache.org/ (accessed on 25 May 2025). The present demonstration implementation employs MinIO https://min.io/ (accessed on 25 May 2025) as the object storage solution, which functions as a data lake in this context even though the architecture calls for using a data lake to store the research data produced by the project partners.

A natural language question-answering system and other web-based data retrieval services are made possible by METRIQA’s decision support mechanism, which is introduced in the *Components layer*. Additionally, it has decision support modeling notebooks that explore data using scripting languages like Python. Platform as a Service (PaaS), Software as a Service (SaaS), and metadata augmentation are further features at this layer.

METRIQA offers a range of user interfaces (UIs) at the *Application layer* that let users engage with the platform. APIs (application programming interfaces) make it easier for customers and suppliers to exchange data.

*Cross-layer* components include the blockchain, security, and data space components. In particular, the data space component guarantees the data space’s correct operation in compliance with the International Data Spaces (IDS) [35] specifications, while the blockchain is coupled with traceability services.

Any supply chain may implement the METRIQA architecture, which was first developed for agri-food supply chains, and where transport actors are already included.

## 5. Design and Development of a Live Demo for Interoperable Traceability

In this section, we introduce a demonstrator based on Use Case 3 from Section 3. The demonstrator is currently under development and serves to illustrate the concept of interoperable traceability. It provides a detailed representation of specific elements within a food supply chain, focusing in particular on the transition from production to processing (including slaughter) of a cow in Italy.

**Figure 2:**
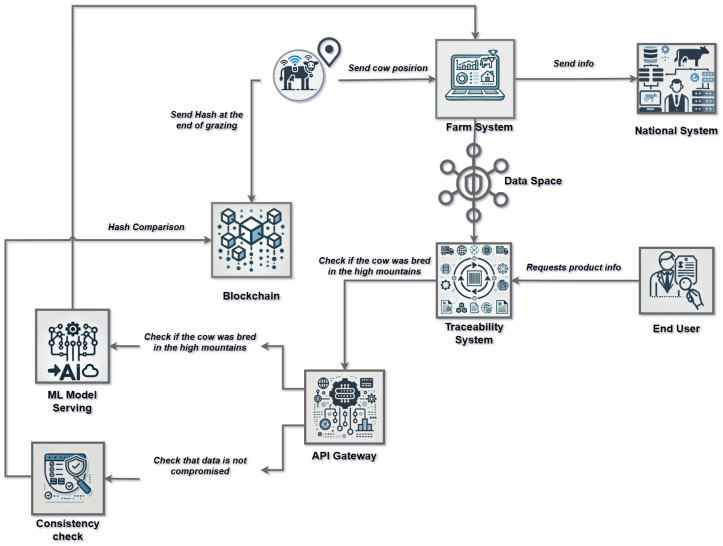
The system architecture of our live demonstrator integrates an IoT client, a private Ethereum Virtual Machine, a connector to the International Data Space (IDS), and tools for data analytics and machine learning.

The functional components of the demonstrator are outlined in Figure 2 and described in detail below.

The data producer, i.e., the cow farmer, is the initial focus. An *IoT collar* that is connected to an IoT client and has a GPS sensor is one of the essential parts of the system. A local database called the *Farm System* (a local information system) contains the raw location data, the owner’s ID, and the ID of the particular cow. As part of the interoperable food supply chain, the cattle farmer plans to sell one of his cows to a slaughterhouse. To make identification easier, the butcher needs to employ a specialized program, like a smartphone app, that can scan the QR code on the collar. Three different kinds of data flows exist:

(i) The federated data space component receives the GPS time series (described in Section 5.3).
(ii) The time series of GPS data is converted into a cryptographic hash, which is a piece of information that supports data integrity, and transmitted to a blockchain smart contract (described in Section 5.2).
(iii) This transaction is outside the scope of this presentation, but in Italy, any change in the ownership of cattle must be recorded to the national database (ital. Banca Dati Nazionale, or BDN).

The METRIQA platform’s federated data space component was created to enable controlled and safe data transfer between connectors inside the data space. Deploying essential components, facilitating participant communication, and verifying data flow scenarios are all part of this procedure. Data sovereignty is maintained while data sharing is made possible by the *connector* to the data space.

The *Big Data Analytics (BDA)* component, acting as the data consumer, performs several key steps to prepare historical data for machine learning applications.

The cow’s movements are tracked by the *Machine Learning* block, which also highlights any instances in which the cow was not grazing in its assigned region. In a similar vein, the *Consistency Check* highlights any problems and performs blockchain-based ongoing data integrity verification. Lastly, using the *API Gateway*, the *Traceability System* block gathers all pertinent data from the *Machine Learning* and *Consistency Check* blocks, allowing it to reply to customer product requests via an additional QR code.

### 5.1. The METRIQA IoT Support for the Demo

A cow’s precise location can be determined using an Internet of Things smart collar that has GPS connectivity. Periodically, roughly every 15 min, the GPS data are gathered and transmitted to the data space via the connector (see Section 5.3). Ultimately, the cow’s GPS history and the farmer’s and cow’s identifying information (i.e., production statistics) are encoded into a cryptographic hash (SHA-256). A private key and recursively length-prefixed (RLP) encoding in binary format are used to sign this transaction. The cloud node that houses the smart contract receives the resultant data after that (see Section 5.2).

To test our demonstration, we decided to emulate the IoT client by using Markov chain models to simulate the evolution of GPS positions (such as shelter, exploration, and foraging) as described in [43,44].

### 5.2. The METRIQA Blockchain for the Demo

The METRIQA platform combines a data space component that controls the information flow between data producers and consumers with a cross-layer blockchain that offers a programmable decentralized ledger. It is clear from the nature of the two METRIQA components that the blockchain component is meant to act as the sole store of data that need to be accessible to all users of the blockchain at all times. On the other hand, all additional data interchange is to be managed by the data space component.

The blockchain implementation under consideration in the given use case is *permissioned*. This indicates that only authorized users (i.e., accounts) with specific roles, each linked to a designated *entity* (a user, a device, etc.), are able to access it. This is required to guarantee the stored data’s legitimacy and non-repudiation. Every entity has a credential that only its owner can use to carry out actions on the entity’s behalf. The accounts enable entities engaging with the blockchain to maintain pseudo-anonymity. This indicates that while the entity linked to each account is not automatically saved in the blockchain, all its communications with the DLT can be audited. Although it is necessary for the use case under consideration, the relationship between accounts and entities may or may not be made available. Three Hyperledger Besu nodes make up the DLT implementation; this blockchain adheres to the Ethereum Virtual Machine and employs the proof-of-authority-based Clique consensus mechanism.

By defining a *smart contract*, i.e., software built on top of the blockchain network that controls the updates of the ledger, the objectives of the use case under consideration are achieved. To be more precise, the smart contract exposes a collection of interfaces that enable its updating (the *functions*) and store data (the *state*). Furthermore, a smart contract allows you to specify which accounts can communicate with it and in what ways. The smart contract, called *Demo-Contract*, is used for the use case in question. It records a hash (a fixed-size string of bytes specific to the input data) related to the historical data of each cow’s pasture and, on top of that, the ownership changes of a cow. More precisely, the smart contract only saves the hash linked to the cow’s pasture data for a number of reasons: The owner of the data has control over which entity is permitted to access them through the data space; it is not necessary to ensure that the data will be available; and the quantity of data that must be stored on a distributed ledger should be kept to a minimum. Ownership changes, on the other hand, are data that need to be accessible to data consumers, which is why the distributed ledger stores them. Keep in mind that the distributed ledger by default offers pseudo-anonymity; therefore, in order to correctly reconstruct all ownership transactions, the association between the account and entity (owner) must be made public.

Every account that communicates with the DEMO-contract is assigned a *role*. Four roles—NoPrivilege, Administrator, Actor, and Device—are defined to grant various privileges to various users. Every account that interacts with the DEMO-contract is given the default role of NoPrivilege. The initial Administrator can extend the Administrator position to additional accounts. The Administrator role is assigned to the Contract Deployer, which is the account that distributes the smart contract to the DL. In this example, all accounts can have responsibilities assigned to them by the administrators. Any account under human control is linked to the Actor role. These accounts can transfer ownership of the cattle they own and operate the gadgets linked to them under the DEMO contract. Data regarding the cow’s pasture can only be registered by the Device role, which is linked to all IoT devices that communicate with the smart contract. The role linked to each account, the ownerships linked to each registered cow, and the relationship between devices, cows, and the present owner are all tracked by the DEMO contract state. Preconditions, or prerequisites that must be fulfilled in order to perform a function, can be linked to any function. This enables the definition of access control, i.e., which accounts are permitted to carry out specific tasks and which state requirements need to be fulfilled in order to update the ledger. For instance, only the Administrator account can carry out the role assignment feature, only the Actor account can register cows and pair them with devices, and only the connected Device can register cow pasture data.

Both the sender and the recipient must recognize the transfer in order for the transfer of ownership to be functional; in other words, the transfer is not finalized until the recipient certifies the transfer of ownership. Data about cow pasture integrity are tracked as events rather than being directly kept in the contract states. Regarding smart contracts, a *event* is a mechanism that enables the contract to convey that a particular event has occurred on the blockchain and its occurrence is recorded in the DL (outside of the contract state). This means that every account that communicates with a smart contract can access every event that has been released since the contract was distributed. This is an alternate method of storing data related to a smart contract that enables the offloading of historical data from the contract itself. The DEMO-contract is alone in charge of controlling whose accounts are permitted to have the cow’s pasture-related information and making sure that a hash linked to a timestamp and a cow is stored correctly. Properly creating the data to be saved in the DL is the responsibility of the software component engaging with the smart contract.

It is helpful to know that *transactions* is used for all requests to update the ledger. A consensus method controls the distributed execution and acceptance of these transactions. A signed data packet that modifies the blockchain’s state is referred to as a transaction in blockchain terminology. In the DL network, it is the main method of starting actions, particularly when interacting with a smart contract. A *Node.js* software component called *Interaction Component* has been created to make it easier to interact with the DL and the DEMO-contract. Three primary services are offered by the Interaction Component: it makes it possible to activate every feature on the DEMO-Contract; it replicates how a smart collar would work to track and maintain the integrity of a cow’s pasture data (see Section 5.1); and in order to confirm the accuracy of the cow’s pasture data, it makes a remote interface available. Through a REST API, the Interaction Component enables remote verification of the accuracy of the pasture data linked to a particular cow.

### 5.3. The METRIQA Data Space for the Demo

METRIQA’s data space implementation complies with the IDS specification [45] and includes all of its essential elements. Implementing the scenario of a farmer as a Data Provider sharing GPS data collected by an IoT device (a cow collar) with a data analytics system (as a Data Consumer) to gather data and train an ML model (as in Use Case 3 presented in Section 3), these data space components have been deployed and tested to validate communication between participants.

By automating container orchestration and guaranteeing the METRIQA IDS’s high availability and efficient resource usage, the components are set up inside a Kubernetes https://kubernetes.io/ (accessed on 25 May 2025) cluster to provide application scalability and resilience. The desire to streamline deployment and administration procedures, enable continuous integration and delivery, and quicken development cycles are further factors driving Kubernetes adoption. Within the cluster, MinIO https://min.io/ (accessed on 25 May 2025) serves as an object storage solution, allowing data access via the connectors. In our demonstrator, we have implemented two connectors: a provider and a consumer. After the Data Owner registers the data as an Offered Resource on the connection, the provider retrieves the data from MinIO.

The connector dubbed TRUE (TRUsted Engineering) Connector (https://github.com/Engineering-Research-and-Development/true-connector (accessed on 25 May 2025)) developed by some of the authors from *Engineering Ingegneria Informatica S.p.A* (https://www.eng.it/en (accessed on 25 May 2025)) is open-source and, on top of that, part of the FIWARE catalog. Because the Execution Core Container and Data App are internally separated, this connector complies with the most recent IDS specifications (such as IDS Info Model 4) and is easily adaptable to a variety of circumstances. It is completely flexible in terms of data formats and protocols (HTTP, HTTPS, Web Socket over HTTPS, and IDSCPv2) and connects with a large number of current IDS services (Identity Provider, Clearing House, Metadata Broker, etc.). The software package consists of a basic data app that may be tailored to fit particular processing and integration needs, as well as a data usage control app to enforce usage regulations. This connector also supports advanced features like *data usage control, attribute-based access control (ABAC)*, and *dynamic policy enforcement* during runtime execution.

### 5.4. Data Analytics of METRIQA in the Demo

Built on cutting-edge frameworks and open-source technologies, the Big Data Analytics Platform (https://home.alidalab.it/ (accessed on 25 May 2025)) is a data science and machine learning solution for creating, implementing, operating, and tracking big data analytics (BDA) workflows in batch and streaming modes.

It makes use of Kubernetes’ resource management capabilities to enable scalable compute and storage. From data ingestion to data preparation, analysis, and publication, the platform provides an expandable catalog of BDA services. The platform offers a web-based interface for creating BDA applications, connects third-party BDA services, and allows data processing from several sources. By integrating resources like datasets and models with BDA services, users may create workflows for both batch and streaming data scenarios. Through a graphical user interface, the Big Data Analytics Platform facilitates the creation, implementation, and operation of BDA applications. One or more BDA services makes up each BDA application. Every BDA service is an OCI-compliant microservice application that is containerized.

Our proposal, based on the Alida platform, comprises several open-source technologies:

- **Kubernetes**: as a resource orchestrator;
- **Argo Workflows** (https://argo-workflows.readthedocs.io/en/latest/ (accessed on 25 May 2025)): an open-source container-native workflow engine for orchestrating parallel jobs on Kubernetes;
- **Apache Kafka** (https://kafka.apache.org/ (accessed on 25 May 2025)): an open-source distributed event streaming platform;
- **React Flow** (https://reactflow.dev/ (accessed on 25 May 2025)): a component used as the basis for the graphical designer to create pipelines of BDA services.

## 6. Evaluation of the Enabling Technologies for Interoperable Traceability

In this section, we evaluate the enabling technologies adopted for our demonstrator. Specifically, we compare alternative distributed technologies, discuss interconnection with the International Data Space, and review networking models for IoT devices.

### 6.1. Distributed Ledger Technology Selection

The increasing need to ensure transparency, security, and trust throughout agri-food traceability and supply chains has promoted blockchain technology as a promising solution for product traceability. Despite its recognized potential, selecting the most suitable blockchain platform remains an open challenge due to the diversity and ongoing evolution of existing solutions. In this section, we present a comparative assessment of various blockchain technologies by analyzing both qualitative and quantitative key performance indicators (KPIs) specifically relevant to the proposed solution. The following indicators were considered: transaction speed, transaction cost, consensus method, smart contract support, IoT readiness, traceability, regulatory metrics (https://digital-strategy.ec.europa.eu/en/policies/blockchain-standards (accessed on 25 May 2025)), and sustainability over time.

Based on these indicators, we analyzed the most prominent blockchain technologies in the literature to assess their suitability for scenarios such as the one described in this article. This analysis will guide further implementation of a version of the platform with a higher technology readiness level (TRL). For brevity, we provide a summary of our findings.

**Ethereum**: Ethereum is the first blockchain platform to support general-purpose smart contracts written in Solidity, introduced in 2015 through the Ethereum Virtual Machine (EVM). It aims to create an open-source platform for decentralized applications (dApps). Initially based on the Proof-of-Work consensus algorithm, Ethereum transitioned to a Proof-of-Stake algorithm in September 2022, reducing its energy requirements. Ether is the second-largest cryptocurrency by market capitalization, demonstrating its strong and enduring appeal among investors and users. In terms of decentralization, the current estimated number of nodes exceeds 12,000 (https://nodewatch.io/ (accessed on 25 May 2025)). Ethereum and EVM-based blockchains have seen wide adoption in industrial applications, including traceability, due to several compelling factors such as a rich availability of smart-contract libraries, frameworks for blockchain interaction, and robust oracle platforms. Additionally, Ethereum has the largest developer community, making it highly attractive in terms of sustainability over time. However, quantitative indicators reveal drawbacks of the mainnet: low transaction throughput (around 15 transactions per second) and high transaction costs. Integration with IoT is facilitated by the availability of numerous libraries.

**Algorand**: Launched in 2019, Algorand’s main proposition is its Pure Proof-of-Stake consensus mechanism. However, its developer community is significantly smaller than that of EVM-compatible platforms, which affects the availability of software libraries for decentralized application development. Currently, the Algorand network has more than 6000 nodes (https://g.nodely.io/d/network/network?orgId=1&from=now-6h&to=now&timezone=browser (accessed on 25 May 2025)) and offers higher transaction throughput and much lower transaction costs than the Ethereum mainnet (https://metrics.algorand.org/#/protocol/ (accessed on 25 May 2025)).

**Solana**: Launched in 2020, Solana combines the classic Proof-of-Stake with the innovative Proof-of-History consensus algorithm. It has seen rapid adoption due to features that promote sustainability over time. The programming language used for smart contract development is Rust, which brings a strong developer community and facilitates IoT platform integration. However, Solana’s blockchain shows a lower degree of decentralization (around 6000 nodes https://solanacompass.com/statistics/decentralization (accessed on 25 May 2025)) but offers high performance in terms of transaction throughput (more than 4000 transactions per second) and low transaction costs https://solscan.io/analytics (accessed on 25 May 2025). Solana has experienced outages in recent years, raising concerns about its reliability and sustainability.

**IOTA**: Launched in 2016, IOTA was designed specifically for the Internet of Things (IoT), aiming to provide a highly scalable, feeless, and secure data transfer protocol. Supported by the IOTA Foundation and a robust community, its DAG-based blockchain has been used in various traceability scenarios [46]. However, IOTA is undergoing significant changes, including updates to its consensus algorithm [47] and network structure, enabling new consensus mechanisms and smart contract programmability in both Layer 1 and Layer 2 using Move and EVM/Solidity, respectively. While these changes set the stage for better adoption, they raise questions about its readiness for projects requiring rapid deployment.

**Polygon PoS**: Polygon is a Layer 2 scaling solution designed to significantly improve scalability, reduce transaction costs, and enhance the speed of the Ethereum network. It ensures interoperability among different blockchains, enabling effective and fast communication between Ethereum-compatible networks. Polygon supports up to 65,000 transactions per second with very low costs, often below 0.01 per transaction, using the Proof-of-Stake (PoS) consensus mechanism. It is fully compatible with the Ethereum Virtual Machine (EVM), allowing easy migration of dApps and smart contracts from Ethereum without substantial modifications. Polygon’s ecosystem offers various scaling technologies such as sidechains, plasma chains, zk-Rollups, and Optimistic Rollups. Its compatibility with a wide range of tools, libraries, and frameworks makes it an attractive and accessible platform for developers and businesses.

The results of our qualitative and quantitative analysis is reported in Table 2. It is clear that Polygon represents an excellent choice for the public blockchain, indicating its complete satisfaction across all indicators, due to its EVM compatibity and sustainability, high transaction speed, and low transaction costs. Conversely, the Ethereum public chain is limited by the partial satisfaction of transaction speed and costs. It is important to note that, considering the open-source nature of Ethereum’s code, creating private instances of the chain, for example, a consortium blockchain based on the Proof-of-Authority consensus algorithm, modifying related parameters, and implementing a periodic checkpointing mechanism on the Ethereum public blockchain could enhance the performance and reduce costs, offering, at the same time, a proper solution in terms of traceability.

### 6.2. Data Space Integration

The International Data Spaces (IDS) [48] framework provides a reference architecture for establishing *sovereign, standardized, and secure data exchange* in trusted business ecosystems. Rooted in European data sovereignty principles, IDS enables data holders to retain full control over how their data is accessed, shared, and used by other entities—ensuring that interoperability does not compromise ownership or governance rights.

At the heart of this architecture is the *IDS Connector*, the core software component that enables technical integration between internal IT systems and the broader IDS infrastructure. Each connector facilitates data transactions according to predefined policies and usage constraints, acting as a gatekeeper that enforces *compliance with IDS specifications* [48]. It supports essential features such as *policy negotiation, contract generation, and secure communication*, and ensures that all participants are authenticated and authorized via certification mechanisms like the *dynamic attribute provisioning service (DAPS)*.

In addition to the TRUE connector adopted in our platform, several other IDS-compliant connectors are relevant, each optimized for different scenarios:

- *Dataspace Connector (DSC)*: developed by Fraunhofer ISST, widely adopted, and supporting strong policy enforcement mechanisms.
- *Eclipse Dataspace Connector (EDC)*: backed by the Eclipse Foundation, with a focus on *Gaia-X* interoperability and extensibility.
- *Trusted Connector*: designed by Fraunhofer AISEC, tailored for IoT-edge deployments where resource constraints and trust guarantees are critical.

The choice of connector must reflect specific operational needs, including scalability, policy complexity, data sensitivity, and integration effort. Once deployed, proper *configuration steps* include the following:

- Interfacing the connector with *existing information systems*;
- Defining *access and usage policies* to ensure *data sovereignty*;
- Registering with *identity and certification authorities* (e.g., DAPS and certificate authorities) to ensure *secure participant authentication and authorization*.

Access and usage policies are regulated through an agreement. More specifically, two parties, typically a Data Provider and a Data Consumer, follow a structured, standardized process that ensures data sovereignty, legal compliance, and technical interoperability [48]. The process begins when the Data Provider issues a Contract Offer describing the conditions under which its data will be made available. The Data Consumer’s IDS Connector sends a Contract Request to the Data Provider’s IDS Connector. The Data Provider’s IDS Connector validates the request for syntax, content, and digital signature. If accepted, a Contract Agreement is created and signed by both parties. The agreement is enforced by the IDS Connectors, which monitor and control data access and usage according to the contract’s terms.

The IDS framework integrates legal, technical, and organizational safeguards. Legal agreements within IDS ensure compliance with regulations such as GDPR, intellectual property laws, and sector-specific requirements. Usage policies are defined and enforced through policy classes and machine-readable contracts, specifying restrictions on data usage (e.g., purpose limitation, time constraints). The IDS Reference Architecture Model (IDS-RAM) and the IDSA Rulebook provide the governance, technical standards, and compliance frameworks for secure, trusted data exchange.

### 6.3. IoT

In the literature, four main communication paradigms have been widely recognized: exclusive pair, publish–subscribe, request–response, and push–pull. The exclusive pair (peer-to-peer) model enables direct, low-latency communication between IoT devices and blockchain gateways. While suitable for isolated environments such as on-farm machinery or point-to-point device coordination, it lacks scalability and robustness in complex supply chains where decentralization and interoperability are essential. The publish–subscribe paradigm is increasingly adopted in IoT-DLT integration. It decouples data producers (e.g., sensors, RFID readers) from consumers (e.g., blockchain interfaces), enabling event-driven architectures. This model is particularly well-suited for real-time monitoring in agricultural logistics, allowing DLT systems to subscribe only to relevant events, optimizing bandwidth and ensuring efficient traceability. The request–response model is effective for querying DLT-provenance information or triggering specific blockchain actions on demand (e.g., product verification at retail checkpoints). While simple and synchronous, it introduces latency in telemetry-heavy applications and is less scalable for continuous data flows. The push–pull approach offers flexibility in scheduled or conditional data acquisition, useful in scenarios where batch processing or historical data analysis is preferred before committing records to the blockchain. However, it may fail to capture critical real-time events, reducing trust guarantees in fast-paced logistics.

Each use case must be carefully analyzed to formulate an optimal solution, which often consists of a well-orchestrated combination (a “cocktail”) of different communication models, selected and tuned according to application-specific requirements and operational constraints.

Beyond the communication model, networking choices are equally critical (see Table 3). While high-power wide area networks (HPWANs) offer broad transmission range, they pose significant limitations in logistics scenarios where assets are continuously moving. HPWAN solutions often require fixed infrastructure and high energy consumption and are less suitable for low-cost, mobile, or battery-powered IoT devices typical in agriculture and livestock supply chains. Low-power wide area networks (LPWANs), such as LoRaWAN, are better suited for mobile logistics applications due to their low energy requirements, long-range coverage, and low deployment cost. However, LoRa may suffer from low data rates and interference in dense environments. NB-IoT (Narrowband IoT) emerges as a promising alternative, combining wide coverage and low energy consumption, with added advantages in supporting mobile nodes and seamless integration with existing cellular networks. The selection of networking technologies must, thus, consider not only energy and cost constraints but also the need for mobility support, reliability in data delivery, and compatibility with DLT-based traceability architectures.

## 7. Discussion and Conclusions

The implementation of interoperable traceability in the agri-food supply chain, as exemplified by the METRIQA platform, highlights the significant progress achieved and ongoing challenges that must be addressed in this field. METRIQA represents a major advance in technology by unifying IoT, blockchain, and data space. The integration of these systems is pivotal in facilitating real-time data capture, secure storage, and seamless exchange. These processes are essential for ensuring the transparency and integrity of the supply chain. Blockchain technology provides an immutable and transparent ledger that fosters trust among stakeholders. Its decentralized nature minimizes the risk of fraud and enhances the credibility of shared information.

While the integration of IoT nodes and DLTs is fundamental to making primary production supply chains trusted, it is equally important to invest in two key aspects for the future: the communication model among system modules and the networking policies adopted to support such integration.

However, achieving interoperability across heterogeneous systems and platforms remains a significant challenge. Variations in data formats, communication protocols, and technical standards require sophisticated solutions to enable consistent and seamless data exchange. To apply METRIQA in other industries, a few adjustments are required. Firstly, custom sensor configurations and device interfaces must be tailored to domain-specific equipment. Secondly, regulatory extensions must be made to address “good practice” guidelines or ISO 22005 compliance. Thirdly, proper data connectors must be designed to allow data sharing and exchange functionalities, aiming to integrate various data sources regardless of their internal technologies and structures. The establishment of effective governance frameworks is critically important in managing this interoperability. While highly coordinated supply chains can easily adopt such frameworks, smaller companies may struggle to implement them without compromising their independence.

The demonstrator highlights practical applications of interoperable traceability in contexts such as wine production, automated sensor data collection, and livestock monitoring. These scenarios illustrate the tangible benefits of the proposed measures, including enhanced operational efficiency, better product quality, and increased consumer confidence. Additionally, the application of machine learning to analyze sensor data enables the generation of actionable insights, turning raw data into valuable information that supports informed decision making, thereby boosting both efficiency and sustainability. All of these highlights extend to the transportation process.

This study affirms that interoperable traceability is an achievable, practical, and effective strategy for improving transparency, efficiency, and trust across agri-food supply chains. Through the integration of IoT, blockchain, and data spaces, the METRIQA platform offers a comprehensive framework to achieve these objectives.

The platform has been developed within the AGRITECH project and is currently in its final stage of deployment to external stakeholders. In the coming months, we expect to obtain user experience data as services are exposed to external users, as planned in the project roadmap.

## Figures and Tables

**Table 1:** Most mature dataspace connectors in public repositories that are interoperable with IDS. Accessed on 25 May 2025.

Name	Repository	TRL	Open-Source
AI.SOV	https://gitlab.cefriel.it/groups/ai-sov	7	No
Dataspace Connector (sovity)	https://github.com/sovity/edc-ce	9	Yes
ECI Gatewise		9	No
FIWARE	https://github.com/FIWARE/data-space-connector	6–7	Yes
GATE	https://github.com/gate-institute/DataspaceConnector/tree/main	5	Yes
Kharon (HOLONIX)		7	No
ONENET	https://github.com/european-dynamics-rnd/OneNet	7	Yes
Telecom DIH		8	No
TNO Security Gateway	https://gitlab.com/tno-tsg	8	Yes
TRUE	https://github.com/International-Data-Spaces-Association/true-connector	6	Yes
TRUSTED	https://github.com/Fraunhofer-AISEC/trusted-connector	6–7	Yes

**Table 2:** Results of qualitative and quantitative analysis of blockchain technologies indicating their suitability ((H)igh, (M)edium, and (L)ow) with respect to their support in traceability scenarios.

	Ethereum	Algorand	Solana	IOTA	Polygon PoS
Transaction speed	M	H	H	H	H
Transaction cost	M	H	H	H	H
Consensus method	H	H	L	M	H
Smart contract support	H	M	M	M	H
Traceability	H	H	H	H	H
Regulatory metrics	H	H	M	M	H
Sustainability over time	H	M	M	M	H

**Table 3:** Strengths and weaknesses of IoT networking models in logistics scenarios.

Networking Model	Strengths	Weaknesses
HPWAN	High coverage range; strong signal penetration	High energy consumption; costly infrastructure; poor mobility support
LPWAN (e.g., LoRaWAN)	Low energy consumption; good range; low deployment cost	Low data rate; possible signal collisions; limited QoS
NB-IoT	Good mobility support; low energy; telco-grade reliability; native integration with cellular	Requires telco infrastructure; limited bandwidth per device
Short-range (e.g., BLE, ZigBee)	Low energy; reliable in local clusters (e.g., within warehouse or barn)	Not suitable for long-range logistics scenarios

## Data Availability

The datasets presented in this article are not readily available because the data are part of an ongoing study. Requests to access the datasets should be directed to Corresponding author.
